# AtfA-Independent Adaptation to the Toxic Heavy Metal Cadmium in *Aspergillus nidulans*

**DOI:** 10.3390/microorganisms9071433

**Published:** 2021-07-02

**Authors:** Tamás Emri, Barnabás Gila, Károly Antal, Fanni Fekete, Heungyun Moon, Jae-Hyuk Yu, István Pócsi

**Affiliations:** 1 Department of Molecular Biotechnology and Microbiology, Faculty of Sciences and Technology, University of Debrecen, 4032 Debrecen, Hungary; gila.barnabas@science.unideb.hu (B.G.); fannifekete05@gmail.com (F.F.); pocsi.istvan@science.unideb.hu (I.P.); 2 Doctoral School of Nutrition and Food Sciences, University of Debrecen, 4032 Debrecen, Hungary; 3 Department of Zoology, Eszterházy Károly University, 3300 Eger, Hungary; antalk2@gmail.com; 4 Department of Bacteriology, University of Wisconsin-Madison, Madison, WI 53706, USA; harrison.moon@wisc.edu (H.M.); jyu1@wisc.edu (J.-H.Y.); 5 Department of Systems Biotechnology, Konkuk University, Seoul 05029, Korea

**Keywords:** *Aspergillus nidulans*, AtfA, cadmium, oxidative stress, transcriptomics

## Abstract

Cadmium is an exceptionally toxic industrial and environmental pollutant classified as a human carcinogen. In order to provide insight into how we can keep our environment safe from cadmium contamination and prevent the accumulation of it in the food chain, we aim to elucidate how *Aspergillus nidulans*, one of the most abundant fungi in soil, survives and handles cadmium stress. As AtfA is the main transcription factor governing stress responses in *A. nidulans*, we examined genome-wide expression responses of wild-type and the *atfA* null mutant exposed to CdCl_2_. Both strains showed up-regulation of the *crpA* Cu^2+^/Cd^2+^ pump gene and AN7729 predicted to encode a putative bis(glutathionato)-cadmium transporter, and transcriptional changes associated with elevated intracellular Cys availability leading to the efficient adaptation to Cd^2+^. Although the deletion of *atfA* did not alter the cadmium tolerance of the fungus, the cadmium stress response of the mutant differed from that of a reference strain. Promoter and transcriptional analyses of the “Phospho-relay response regulator” genes suggest that the AtfA-dependent regulation of these genes can be relevant in this phenomenon. We concluded that the regulatory network of *A. nidulans* has a high flexibility allowing the fungus to adapt efficiently to stress both in the presence and absence of this important transcription factor.

## 1. Introduction

Cadmium is a highly toxic, nonessential heavy metal. Although it is regularly found in ores of zinc, lead, and copper, the elevated cadmium levels detected in the environment are of anthropogenic origin in most cases [1,2,3,4]. Atmospheric deposition, water irrigation, and the use of phosphate fertilizers with high cadmium content can all increase cadmium concentrations in agricultural fields and lead to accumulation of this toxic heavy metal in the food chain [1,4]. Deeper understanding of cadmium stress response may help in improving Cd^2+^ biosorption techniques based on microbial biomass [5,6,7,8,9,10] as well as developing new strategies to prevent the accumulation of Cd^2+^ in plants [10,11].

AtfA is a bZIP-type transcription factor of *Aspergillus nidulans* [12,13]. AtfA and its orthologs regulate sexual and asexual development, different stress responses, and secondary metabolism in filamentous fungi [14,15,16,17,18,19]. Not surprisingly, it contributes substantially to the virulence of both the human and plant pathogenic fungi [16,17,18,20,21,22]. Previously we found that the *ΔatfA* strain was more sensitive to oxidative stress induced by H_2_O_2_, menadione sodium bisulfite, *terc*-butylhydroperoxide, or diamide than the reference strain [23]. Transcriptomic studies also demonstrated that AtfA plays an important role in the regulation of oxidative stress: Deletion of its gene affected the transcription of a large number of genes during stress treatments [23,24,25]. In addition to the large number of genes affected, the stressor specificity of the AtfA-dependent genes and similar numbers from up- and down-regulated genes suggest that most affected genes are indirectly regulated by AtfA [24]. Elements of the phosphorelay response regulator systems (e.g., *fphA*, *nikA*, *phkA*, *srrB*, *srrC*, *sskA,* or *tcsB*) are among the few genes that showed consequent down-regulation in the *ΔatfA* mutant in comparison to the reference strain [23,24,25]. The strong AtfA-dependence of these regulatory genes raises the possibility that they are under direct control of AtfA. This would explain why deletion of *atfA* had a strong effect on the transcriptome.

Here we used a transcriptomics-based approach using RNAseq to elucidate the CdCl_2_-induced stress response in *A. nidulans* as a model organism. In addition to the reference strain, we also recorded the cadmium stress response of a *ΔatfA* mutant. Investigating two different strains helped us to better identify key genes and processes of Cd^2+^ tolerance and also allowed us to better understand the role of AtfA in the regulation of stress responses.

## 2. Materials and Methods

### 2.1. Strains and Culture Conditions

The *A. nidulans* THS30 (*pyrG89; AfupyrG^+^; pyroA4; pyroA^+^*) as reference strain and TNJ92 (*pyrG89; AfupyrG^+^; pyroA4; ΔatfA::pyroA*) as a *ΔatfA* gene deletion mutant [23] were used in this study. Strains were maintained on Barratt’s minimal medium agar plates at 37 °C [26]. Conidia freshly collected from 6 d cultures were used for inoculation in all experiments.

For submerged cultivation, 100 mL Barratt’s minimal broth (in 500 mL Erlenmeyer flasks) was inoculated with 100 × 10^6^ conidia and was incubated at 37 °C and at 3.7 Hz shaking frequency for 16.5–26 h. Before stress treatment (0.2 mM CdCl_2_, at 16 h) the biomass of cultures were set to equal levels as described earlier [23].

### 2.2. Recording Growth, Measuring GSH and GSSG Contents, 2′,7′-Dichlorofluorescein-Assay (DCF-Test)

Mycelia from 10 mL fermentation broth were filtered and dried at room temperature. The increase in the dry cell mass (DCM) values between 16 h and 26 h were calculated and used to characterize the growth of CdCl_2_-treated and untreated cultures.

Mycelia samples taken at 18.5 h and treated with 5′-sulfosalicylic acid [27,28] were used for the determination of (reduced) glutathione (GSH) and oxidized glutathione (GSSG) content with the DTNB-glutathione reductase assay [29] as well as for DCF-test to record changes in the redox homeostasis of the cultures [30].

### 2.3. Measuring Specific Enzyme Activities

Specific catalase and superoxide dismutase (SOD) activities were measured by rate assays [31,32] with cell-free extracts prepared by X-pressing [33]. The same cell free extracts were used to detect Cys synthase activities by the colorimetric method of Warrilow and Hawkesford [34] with O-acetyl-serine and Na_2_S as substrates. Protein content of the samples was measured with Bradford reagent. Chitinase, N-acetyl-glucosaminidase, and β-glucosidase activities were determined from fermentation broth with 4-nitrophenyl-β-D-*N*,*N*′,*N*′′-triacetylchitotriose,4-nitrophenyl-*N*-acetyl-β-D-glucosaminide and 4-nitrophenyl-β-D-glucoside as substrates, respectively, according to Pusztahelyi et al. [35]. Due to low enzyme activity values, the fermentation broth was concentrated tenfold before the hydrolase activity measurements using Amicon Ultra (Merck, Budapest, Hungary) centrifugal filters.

### 2.4. Reverse-Transcription Quantitative Real-Time Polymerase Chain Reaction (RT-qPCR) Assays

Lyophilized mycelia were used for isolation of total RNA as described previously by Chomczynski [36]. RT-qPCR assays were carried out as detailed in Kovács et al. [37], with the primer pairs listed in Table S1 and using the *actA* gene as reference. Relative transcription was characterized with either ΔCP or ΔΔCP. ΔCP = CP_r_ − CP_t_ where r, t, and CP stand for the reference gene, target gene, and the crossing point value detected in the RT-qPCR assays, respectively. ΔΔCP_treatment_ = ΔCP_treated culture_ − ΔCP_untreated culture_ and ΔΔCP_mutation_ = ΔCP_mutant_ − ΔCP_reference strain_.

### 2.5. High-Throughput RNA Sequencing

Four different cultures with three biological replicates were studied. For “untreated cultures” of the reference strain (THS30) and the *ΔatfA* mutant (TNJ92), Barrat’s minimal broths were inoculated with conidia of the THS30 or the TNJ92 strains as described above and were incubated for 16.5 h at 37 °C and at 3.7 Hz shaking frequency. For “CdCl_2_-treated cultures” of the same strains, Barrat’s minimal broths were inoculated with conidia of the THS30 or the TNJ92 strains and were incubated for 16.5 h at 37 °C and at 3.7 Hz shaking frequency. These cultures were treated with 0.2 mM CdCl_2_ at 16 h.

Total RNA were isolated from cultures independent to those used for RT-qPCR assays. RNA sequencing (from library preparation to generation of fastq.gz files) were carried out at the Genomic Medicine and Bioinformatics Core Facility, Department of Biochemistry and Molecular Biology, Faculty of Medicine, University of Debrecen, Debrecen, Hungary. RNAseq libraries were prepared with TruSeq RNA Sample preparation kit (Illumina, Praha, Czech Republic) according to the manufacturer’s protocol. Each library pool was sequenced (single-read 75 bp sequencing on an Illumina HiScan SQ instrument; Illumina, San Diego, CA, USA) in one lane of a sequencing flow cell, and 11–16 million reads per sample were obtained. Reads were aligned to the genome of *A. nidulans* FGSC A4 with hisat2 (version 2.1.0) [38] using the following genome and genome features files (GFF) http://www.aspergillusgenome.org/download/sequence/A_nidulans_FGSC_A4/archive/A_nidulans_FGSC_A4_version_s10-m04-r12_chromosomes.fasta.gz (accessed on 2 July 2021) and http://www.aspergillusgenome.org/download/gff/A_nidulans_FGSC_A4/archive/A_nidulans_FGSC_A4_version_s10-m04-r12_features_with_chromosome_sequences.gff.gz (accessed on 2 July 2021), respectively. The percentage of the successfully aligned reads varied between 80–91%. Differentially expressed genes were determined with DESeq2 (version 1.24.0) [39]. RPKM values (reads per kilo base per million mapped reads) used only to visualize transcription activities of selected genes were calculated with the “rpkm” function of the edgeR package [40].

### 2.6. Evaluation of RNAseq Data

“Up-regulated and down-regulated genes were defined as differentially expressed genes (DESeq2; *p* < 0.05) where the log_2_FC > 0 and log_2_FC < 0, respectively. Log_2_FC stands for the number calculated by the DESeq2 software using THS30 as reference strain (TNJ92 untreated cultures vs. THS30 untreated cultures or TNJ92 Cd treated cultures vs. THS30 Cd treated cultures) or untreated cultures as reference conditions (THS30 Cd treated cultures vs. THS30 untreated cultures or TNJ92 Cd treated cultures vs. TNJ92 untreated cultures). Up-regulated and down-regulated genes identified in the CdCl2-treated vs. untreated and in the *ΔatfA* mutant vs. reference strain comparisons were regarded as stress responsive and AtfA-dependent genes, respectively”.

Gene groups containing functionally related genes were defined as follows:

“Antioxidative enzyme” genes: Genes of known or putative catalases, peroxidases, superoxide dismutases, or of the glutathione/glutaredoxin/thioredoxin system collected from the *Aspergillus* Genome Database (AspGD; http://www.aspergillusgenome.org; accessed on 2 July 2021).

“Autophagy” genes, “DNA repair” genes, and “P-type ATPase” genes: Genes with “autophagy”, “role in DNA repair”, or “P-type ATPase” feature, respectively, in their general description according to the AspGD.

“Cd^2+^ efflux pump” genes: Cation transporter genes of “cadmium ion import into vacuole”, “cadmium ion transport”, and “cellular detoxification of cadmium ion” GO terms (AspGD) as well as *ygA* (AN3624, [41]), an ortholog of *A. fumigatus pcaA* (Afu1g16130) putative cadmium transporter [8,10].

“Cell wall biosynthesis and degradation genes as well as cell wall integrity pathway” genes: Genes encoding enzymes of cell wall polysaccharide biosynthesis and degradation as well as of the cell wall integrity (protein kinase C) MAPK pathway according to De Groot et al. [42].

“Cys, Met and GSH metabolism” genes: Genes of GSH synthesis and degradation, Cys and Met biosynthesis, transsulfuration pathway, Met cycle., and Met salvage pathway according to the KEGG Pathway Database (https://www.genome.jp/kegg/pathway.html, accessed on 2 July 2021).

“Cys rich protein” genes: Gene encoding proteins with more than 10% Cys content according to the AspGD.

“ER to Golgi vesicle-mediated transport” genes: Genes of the “endoplasmic reticulum to Golgi vesicle-mediated transport” GO term according to the AspGD.

“Fe-S cluster binding protein” genes: Genes of the “iron-sulfur cluster binding” GO term according to the AspGD.

“Folate cycle” genes and “Glyoxylate pathway”genes: Genes of the folate cycle as well as of the glyoxalate cycle according to the KEGG Pathway Database.

“Glycolysis genes”: Genes described by Flipphi et al. [43].

“Phosphorelay response regulator” genes: Genes of the “phosphorelay response regulator activity” and “osmosensory signaling via phosphorelay pathway” GO terms in the AspGD.

“Ribosome biosynthesis” genes: Genes of the “ribosome biogenesis” *Aspergillus* GO-slim process category (http://www.aspergillusgenome.org/cgi-bin/GO/goTermMapper, accessed on 2 July 2021).

“Secondary metabolism cluster” genes: Manually or experimentally identified secondary metabolite cluster genes according to Inglis et al. [44]. 

“Squalene-ergosterol pathway” genes: Orthologs of *A. fumigatus* squalene–ergosterol pathway genes [45].

“Zinc transporter” genes: Genes of the “zinc ion transmembrane transporter activity”, “zinc ion transport”, and “zinc ion transmembrane transport” GO terms in the AspGD.

The enrichment of genes from the above defined genes groups within the up-regulated, down-regulated, stress responsive, and AtfA-dependent gene sets was tested with the “fisher.test” function (Fisher’s exact test; *p* < 0.05) of the R project (www.R-project.org, accessed on 2 July 2021).

Gene set enrichment analyses with FunCat, GO, and KEGG pathway terms were carried out with the FungiFun2 package (https://elbe.hki-jena.de/fungifun/fungifun.php, accessed on 2 July 2021) using default settings. Hits with adjusted *p* < 0.05 were taken into consideration during the evaluation process.

### 2.7. Promoter Analysis

Putative AtfA binding sites were identified according to Szabó et al. [19] in the promoter of each “Phosphorelay response regulator”, known and putative catalase, “Glycolysis”, “Squalene-ergosterol pathway”, “Folate cycle”, and Asperfuranone cluster genes of *A. nidulans*. In the case of *A. fumigatus*, *A. niger,* and *A. oryzae,* only the orthologs or best hit(s) for the *A. nidulans catA*, *catB,* and the “Phosphorelay response regulator” genes (according to AspGD) were studied. Briefly: The 5′-upstream intergenic sequences were downloaded from the AspGD and transcription start sites (TSS) were determined with the FGENESH tool (Softberry; http://www.softberry.com, accessed on 2 July 2021) in these sequences. ATF/CREB family transcription factor binding sites were searched with the PROMO (version 3.0.2) tool (http://alggen.lsi.upc.es/cgi-bin/promo_v3/promo/promoinit.cgi?dirDB=TF_8.3, accessed on 2 July 2021) using 5% maximum matrix dissimilarity rate. Sites in the regions from TSS-1000 to TSS+50 and from TSS+50 to ATG were counted separately. In the case when identification of TSS failed, only the region from ATG-1500 to ATG was searched for binding motifs. 

## 3. Results

### 3.1. Deletion of atfA Did Not Alter Significantly the Physiology of CdCl_2_-Treated Cultures

**Figure 1:**
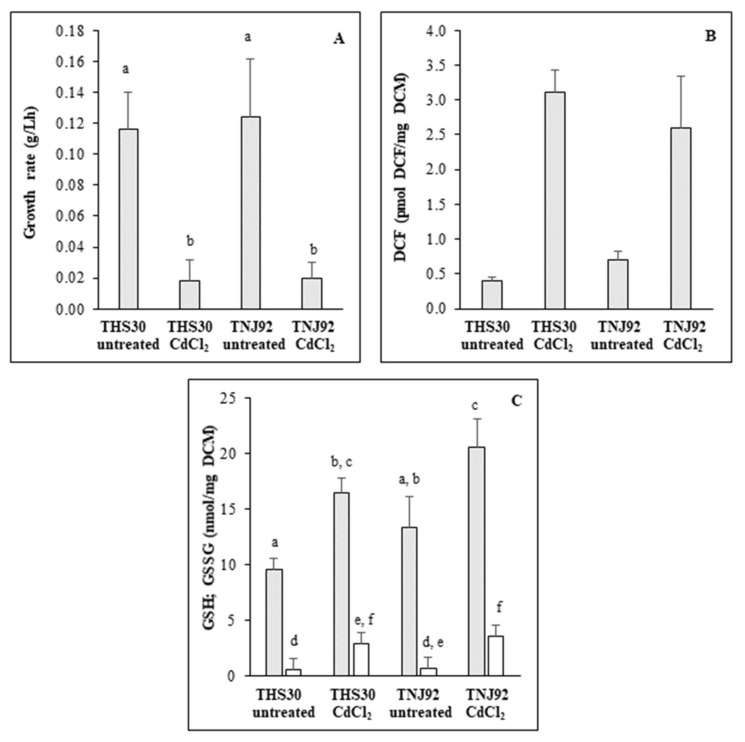
Physiological consequences of cadmium treatment on *A. nidulans*. The *ΔatfA* mutant (TNJ92) and the reference (THS30) strains were treated with 0.2 mM CdCl_2_. Stress tolerance was characterized with the growth rate of the cultures (**A**). Redox imbalance caused by CdCl_2_ treatment was characterized with DCF-test (**B**). GSH (grey) and GSSG (white) content of treated and untreated cultures were recorded with the DTNB-GR assay (**C**). Mean ± SD. Values (n = 3) are presented. According to the two-way-ANOVA the CdCl_2_ treatment had a significant effect on DCM formation (*p* = 0.0000867), DCF production (*p* = 0.000003148) as well as on the GSH (*p* = 0.00004586) and GSSG (*p* = 0.00117) contents of cells. The genotype of the strains had a significant effect only on GSH content (*p* = 0.0097778), but the genotype did not influence the effect of CdCl_2_ treatment (*p* = 0.994) even in this case. Means of the same variable marked with a common letter are not significantly different by the Tukey post-hoc test at the *p* = 0.05 level.

Cadmium treatment inhibited the growth in submerged cultures as expected (Figure 1A). Significant difference between the TNJ92 *ΔatfA* mutant and the THS30 reference strain was not detected (Figure 1A).

Similar results were obtained on agar plates earlier where the relative growth values (ratio of colony diameters of treated and of untreated cultures) of the same two strains were very similar [25]. Cadmium stress significantly disturbed the redox balance in both strains, but there was no significant difference between their behaviors (Figure 1B). In addition, cadmium treatment increased GSH and GSSG contents of mycelia, and the specific SOD (but not catalase) activities displayed a very similar pattern in both strains (Figure 1C, Table 1).

### 3.2. Deletion of atfA Resulted in Marked Changes in Transcriptomes Observed under CdCl_2_ Stress

The transcriptomes of four different cultures (untreated and CdCl_2_-treated cultures of the TNJ92 *ΔatfA* mutant and the THS30 reference strain) were compared using RNA sequencing (RNAseq; Table S2). Transcriptional changes of 24 genes were also determined by RT-qPCR (Table S3) and they showed good correlation with the RNAseq data (Figure S2). According to the principal component analysis (PCA) of the RNAseq data, both the stress treatment and the genotype of the strains markedly affected the transcriptomes (Figure S1).

**Figure 2:**
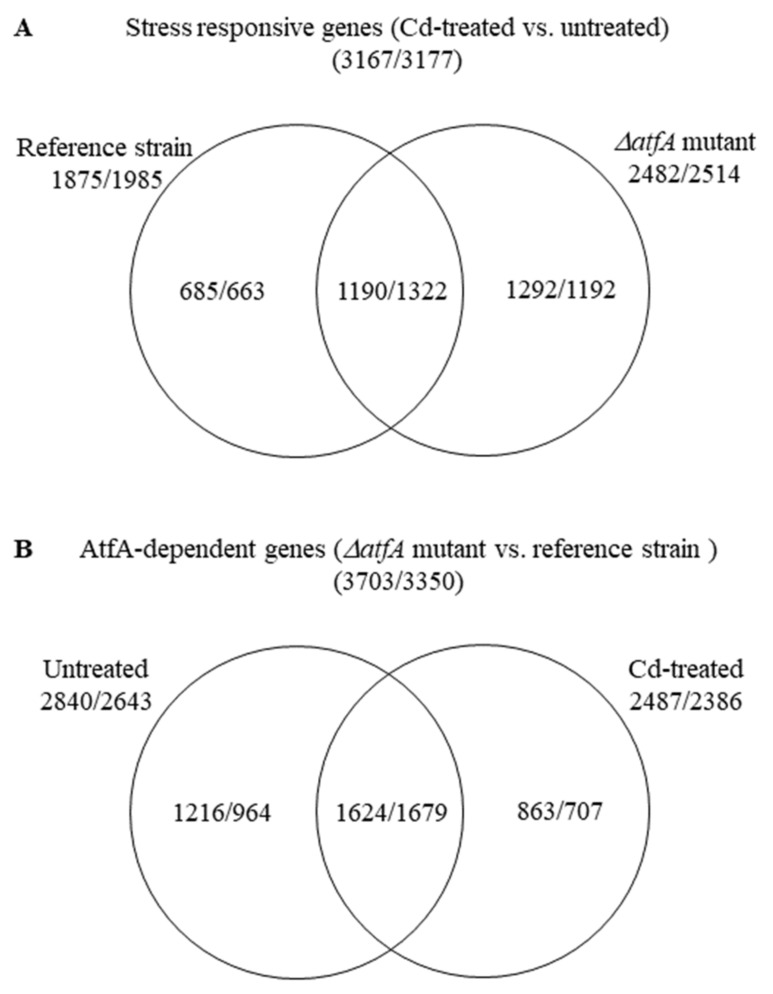
Distribution of stress responsive genes between the THS30 reference strain and the TNJ92 *ΔatfA* mutant (**A**) as well as of the AtfA-dependent genes between the untreated and CdCl_2_-treated cultures (**B**). Figures represent the number of up-regulated/down-regulated genes. For definition of up-regulated, down-regulated, stress responsive, and AtfA-dependent genes. See the chapter titled “2.5 Evaluation of RNAseq data”.

Composition of the differentially expressed gene sets showed that the stress responses of the TNJ92 *ΔatfA* mutant and the THS30 reference strain were different: 62% (685 + 1292 = 1977) of the 3167 up-regulated and 58% (663 + 1192 = 1855) of the 3177 down-regulated stress responsive genes showed altered transcription either in the mutant or in the reference strain only (Figure 2A). The stress treatment also changed the transcriptional difference between the two strains: 56% (1216 + 863 = 2079) of the 3703 up-regulated and 50% (964 + 707 = 1671) of the 3350 down-regulated “AtfA-dependent genes” showed AtfA-dependence only in untreated or only in CdCl_2_-treated cultures (Figure 2B).

### 3.3. CdCl_2_ Stress Regulates Vegetative Growth, Cd^2+^ Efflux System, and Sulfur Metabolism in the Reference Strain

Gene set enrichment analyses were applied to elucidate how the THS30 reference strain adapted to the CdCl_2_ treatment-induced stress. According to them, the stress down-regulated several processes involved in the fast vegetative growth in *A. nidulans*. These included glycolysis, tricarboxylic acid (TCA) cycle, respiration, and sterol biosynthesis (Table 2, Table 3, Tables S4 and S5). Interestingly “Ribosome biogenesis” genes were significantly enriched in both the up-regulated and the down-regulated gene sets (Table 3 and Table S5), suggesting a remodeling rather than bulk unidirectional changes in protein synthesis under stress.

Out of the potential Cd^2+^ efflux pumps, *crpA* (AN1317) [9,46], but not *ygA,* was up-regulated (Tables S3 and S5). Apart from *crpA*, two other putative P-type ATPase genes (AN0318 and AN10367) were also up-regulated under cadmium stress (Table S5).

Fungal cell wall can bind a huge amount of Cd^2+^, and chitin can be particularly important in this biosorption [47,48]. Interestingly, cadmium stress did not cause substantial changes in the transcription of genes involved in cell wall biosynthesis or degradation and this was the case with extracellular chitinase, *N*-acetyl-β-D-glucosaminidase, and β-glucosidase activities as well (Table 1 and Table S5).

In *A. nidulans*, little is known on metallothioneins (Cys-moiety rich, low molecular weight proteins); however, they can be potentially important to detoxify heavy metals including cadmium [46,49]. Out of the genes encoding proteins with more than 10% Cys content, only two genes (AN7290 and AN11757) showed up-regulation (Table S5). Moreover, “Cys rich protein” genes including *crdA*, encoding a putative metallothionein of *A. nidulans* [46], were enriched in the down-regulated gene set (Table S5). Consequently, bulk up-regulation of metallothionein genes is not an important element of cadmium-induced stress response in *A. nidulans*. 

“Glyoxylate pathway” genes, similarly to other genes involved in mitochondrial processes like TCA cycle and respiration, were down-regulated by cadmium treatment, therefore—in contrast to *A. foetidus* [6]—complexation of Cd^2+^ with secreted oxalate is unlikely to contribute substantially to the reduction of Cd^2+^ toxicity (Table 3 and Table S5) under the studied culturing conditions. 

**Figure 3:**
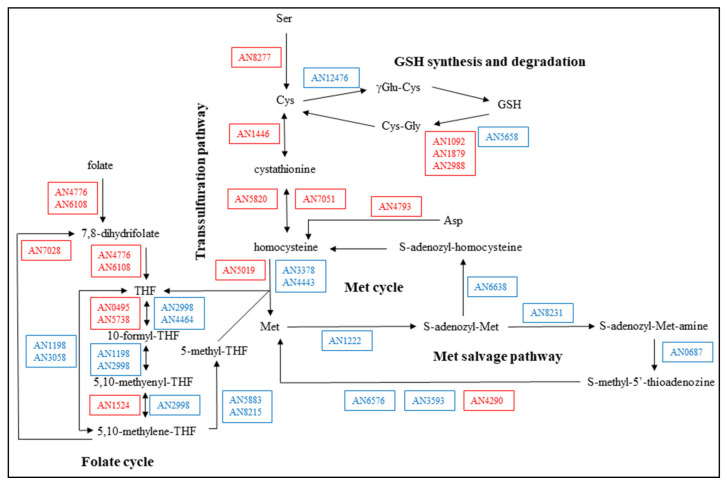
Changes in the Cys and Met metabolism under CdCl_2_ treatment in the *A. nidulans* THS30 reference strain. Up-regulated genes are marked with red, down-regulated genes with blue.

Sulfur metabolism was significantly affected by the stress treatment (Table 3 and Table S5, Figure 3). Surprisingly, according to the observed transcriptional changes, cells increased Cys availability by up-regulating the transsulfuration pathway, GSH degradation, and Cys biosynthesis, and down-regulated the usage of Met via Met cycle and Met salvage pathway (one carbon transfer processes) (Table S5, Figure 3). Consistent with this, specific Cys synthase activities were also significantly increased by the CdCl_2_ treatment (Table 1). Rearrangement (both up-regulation and down-regulation) of folate cycle genes was also observed (Table 3 and Table S5, Figure 3).

Although AN7729 (ortholog of *Saccharomyces cerevisiae ycf1* [50] and AN4517 (ortholog of *Schizosaccharomyces pombe hmt1* [51] are both transmembrane transporter genes putatively involved in the transportation of bis(glutathionato)-cadmium complex into the vacuole, only the former was up-regulated (Tables S3 and S5).

Enrichment of “Fe-S cluster binding protein” genes within the down-regulated gene set as well as the enrichment of proteosomal degradation and autophagy genes within the up-regulated gene set were also observed (Table 3 and Table S5).

Regarding the two most important ROS producing cell organelles, mitochondrium and endoplasmic reticulum (ER), biochemical processes localized to mithochondria (e.g., TCA cycle, glyoxylate pathway, and respiration) were down-regulated (Table 2 and Table S4). In contrast, cadmium stress up-regulated the unfolded stress response together with the “ER to Golgi vesicle transport” genes but down-regulated the “Squalene-ergosterol pathway” genes (Table 2, Table 3 and Table S5). The importance of ER in cadmium stress response as well as the up-regulation of unfolded stress response under cadmium stress was also demonstrated in *S. cerevisiae* [52].

Interestingly the oxidative stress induced by CdCl_2_ treatment (Figure 1B) was not accompanied by bulk up-regulation of genes encoding antioxidative enzymes (Table 3 and Table S5). Some genes (e.g., *sodB*, manganese-superoxide dismutase [53]; *ccp1*, putative cytochrome c peroxidase [54]; *catB* and *catC*, catalases [55]) were up-regulated, while others (e.g., *trxA*, thioredoxin [56]; *gpxA*, putative glutathione peroxidase [54]; *cpeA*, putative catalase-peroxidase [54,55]) were down-regulated (Table S5). These transcriptional changes were accompanied with increased specific SOD activities (Table 1). In contrast, specific catalase activities remained unchanged (Table 1), which can be explained with multiple, differentially regulated catalase genes [55] affecting catalase activities [55]. Similar behavior of the “Antioxidative enzyme” genes (i.e., stress-dependent remodeling of their activity) was observed under oxidative stress induced by carbon starvation earlier [57,58].

Cd^2+^ can substitute Zn^2+^ in proteins, and elevated intracellular Zn^2+^ content can protect against it [59,60]. Interestingly, Zn^2+^ transporters showed down-regulation (Table 3 and Table S5) in our experiments. The down-regulated transport can be the consequence of reduced zinc usage caused by decreased growth (Figure 1A) under cadmium stress. It is also possible that Cd^2+^ enters the cells via Zn^2+^ transporters, therefore down-regulation of these transporters can be important to reduce the infiltration of Cd^2+^ into the cells.

“Excision repair” genes showed significant enrichment within the up-regulated gene set (Table 3 and Table S5) and some other “DNA repair” genes were also up-regulated (Table 3 and Table S5), which is in line with the genotoxicity of Cd^2+^ [61].

In contrast to oxidative, osmotic or carbon starvation stress [23,58] the applied cadmium stress had minor effect on the transcription of “Secondary metabolite cluster” genes (Table 3 and Table S5). A few clusters, however, showed up-regulation (inp cluster) or down-regulation (microperfuranone cluster, AN1242 cluster) (Table 3 and Table S5).

### 3.4. At the Level of Regulated Gene Groups, Deletion of atfA Caused Only Minor Changes in the Stress Response Induced by CdCl_2_

Cadmium stress also down-regulated several processes involved in the fast vegetative growth in the TNJ92 *ΔatfA* mutant. These included TCA cycle, respiration, and sterol biosynthesis (Table 2, Table 3, Tables S4 and S5). Moreover, mitotic cell cycle genes, as well as DNA synthesis and replication genes, were also significantly enriched in the down-regulated gene set of the mutant (Table 2 and Table S4). In contrast to the THS30 reference strain, “Glycolysis” genes were not down-regulated and “Ribosome biogenesis” genes showed bulk down-regulation instead of remodeling (Table 3 and Table S5).

The putative Cd^2+^ efflux pump gene, *crpA*, was up-regulated (Tables S3 and S5) and the *crdA* putative metallothionein gene was down-regulated (Table S5). Cadmium stress did not substantially change the transcription of cell wall homeostasis genes (Table S5); however, cell wall biosynthesis genes were significantly enriched within the stress responsive gene set, suggesting some remodeling. In line with this, minor changes in the extracellular chitinase and *N*-acetyl-β-D-glucosaminidase but not in β-glucosidase activities were also recorded in the *ΔatfA* gene deletion mutant after CdCl_2_ treatment (Table 1). Interestingly, β-glucosidase activities were significantly higher in the cultures of the mutant strain than in the reference strain grown in either cadmium-treated or untreated cultures (Table 1).

In the case of sulfur metabolism genes (Table 3 and Table S5, Figure S3), “Fe-S cluster protein” genes, “Autophagy” genes, and proteosomal degradation genes, changes similar to those observed with the THS30 reference strain were detected in the TNJ92 *ΔatfA* mutant (Table 2, Table 3, Tables S4 and S5). Up-regulation of “Folate cycle” genes, however, was not observed in the mutant (Table 3 and Table S5, Figure S3). Importantly, the specific Cys synthase activities increased after cadmium treatment (Table 1).

Mitochondrium- and ER-related processes behaved similarly in the TNJ92 mutant and THS30 reference strain: In both strains, cadmium stress down-regulated mitochondrium-related processes like TCA cycle, glyoxylate pathway, and respiration (Table 2 and Table S4), and up-regulated genes in the “Protein processing in endoplasmic reticulum” and “ER to Golgi vesicle transport” groups but down-regulated “Squalene-ergosterol pathway” genes (Table 2, Table 3, Tables S4 and S5). Rearrangements in the transcription of “Antioxidative enzyme” genes were also found (Table 3 and Table S5): In the case of the *ΔatfA* mutant, the up-regulated genes included *sodA* [62] and *sodB* superoxide dismutases, *ccp1* putative cytochrome c peroxidase, and *catA* catalase [55], while the down-regulated genes included *trxA* thioredoxin and *trxB* thioredoxin reductase [56] (Table S5). Similarly to the reference strain, CdCl_2_ treatment increased the specific SOD but not the specific catalase activities (Table 1). Consistent with the transcriptional values of most catalase genes (Table S5), the specific catalase activities were significantly lower in the AtfA-mutant than in the reference strain (Table 1).

Zn^2+^ transporter genes also showed down-regulation in the TNJ92 mutant (Table 3 and Table S5). Interestingly, unlike in THS30, “DNA repair” genes were down-regulated and even the “Excision repair” genes did not show significant enrichment in the up-regulated gene set (Table 3 and Table S5).

Bulk up-regulation of “Phosphorelay response regulator” genes was observed only in the *ΔatfA* mutant (Table 3 and Table S5).

Cadmium stress caused only minor changes in the transcription of secondary metabolism genes: Up-regulation of the inp, AN7884 and AN7084 clusters as well as down-regulation of the microperfuranone cluster were observed (Table 3 and Table S5).

### 3.5. Background of the Differences Observed between the Stress Responses of the Two Strains 

In order to understand the nature of the differences between the CdCl_2_ stress responses of the TNJ92 *ΔatfA* mutant and the THS30 reference strain, we investigated the behavior of individual genes in selected gene groups.

#### 3.5.1. Diverging Gene Sets Regulate Same Biological Processes under Cadmium Stress

**Figure 4:**
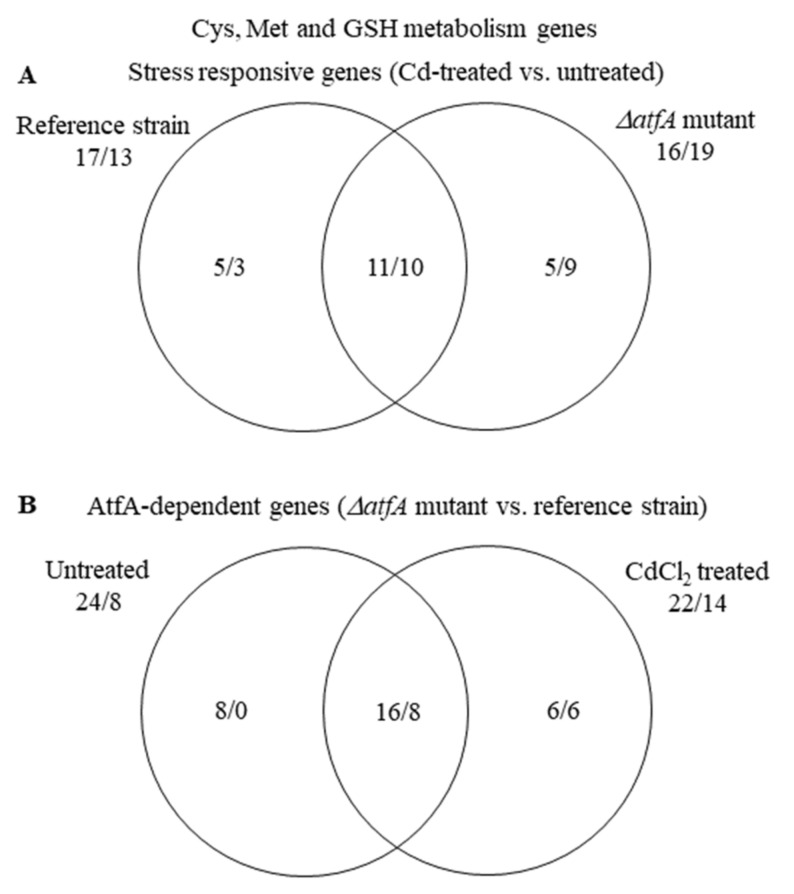
Distribution of stress responsive “Cys, Met and GSH metabolism” genes between the THS30 reference strain and the TNJ92 *ΔatfA* mutant (**A**) as well as of the AtfA-dependent “Cys, Met and GSH metabolism” genes between the untreated and CdCl_2_-treated cultures (**B**). Figures represent the number of up-regulated/down-regulated genes. Further data on this gene group are available in Table S5.

During cadmium stress, the transcriptional changes of the “Cys, Met, and GSH metabolism” genes suggested a very similar conclusion, i.e., cells tried to increase Cys availability, in both strains (Figure 3 and Figure S3, Table S5). The overlap between the stress responsive genes of the two strains in this gene group, however, was only 49% (21 out of 43) (Figure 4). Similarly, the “ER to Golgi vesicle-mediated transport” genes were significantly enriched in the up-regulated gene set in both strains (Table 3 and Table S5), yet their overlap was only 45% (10 out of 22) (Figure S4). Even in the case of the “Cell wall biosynthesis and degradation” genes where bulk up-regulations or down-regulations were not observed in either strain (Table S5), the overlap between the stress responsive genes of the *ΔatfA* mutant and the reference strain was only 39% (24 out of 61) (Figure S5). These data suggest that the two strains initiated similar physiological changes under cadmium stress but they did it with partly different gene sets. It concurs with our previous observations when the effects of two different stressors were investigated on the same strain: We found that both menadione and diamide inhibited the growth of *A. nidulans* THS30 and down-regulated several “Ribosome biogenesis” genes (36 and 30 genes for menadione and diamide stress treatment, respectively), but the overlap (1 gene) was negligible [24].

#### 3.5.2. Differences between the Transcriptomes of Untreated Cultures Led to Different Stress Responses

**Figure 5:**
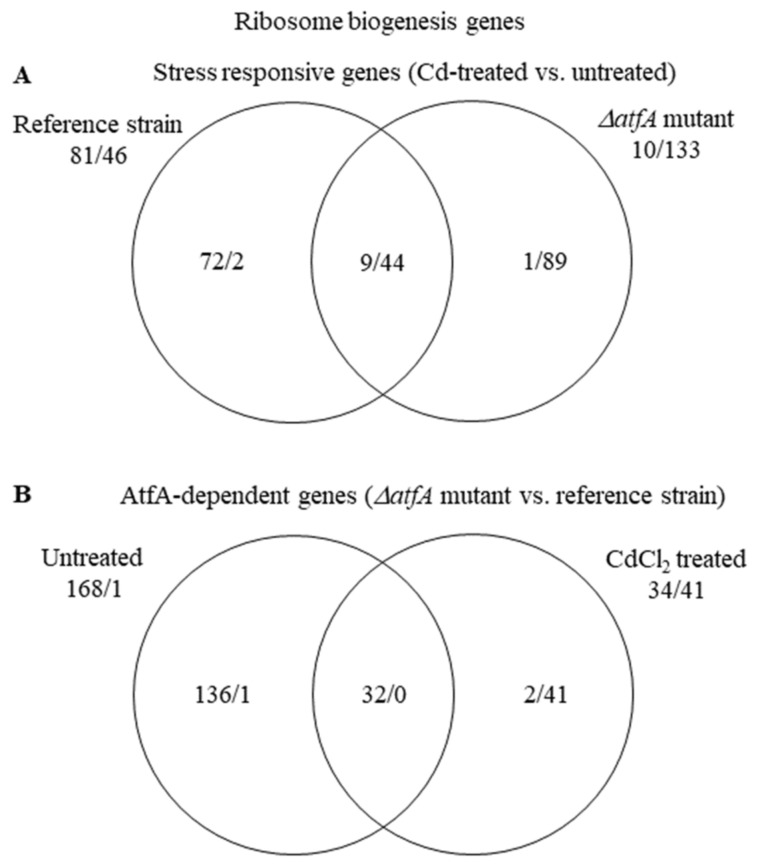
Distribution of stress responsive “Ribosome biogenesis” genes between the THS30 reference strain and the TNJ92 *ΔatfA* mutant (**A**) as well as of the AtfA-dependent “Ribosome biogenesis” genes between the untreated and CdCl_2_-treated cultures (**B**). Figures represent the number of up-regulated/down-regulated genes. Further data on the gene group are available in Table S5.

In the THS30 reference strain, the “Ribosome biogenesis” genes were significantly enriched in both the up-regulated and the down-regulated gene sets, in the TNJ92 *ΔatfA* mutant, however, only in the down-regulated gene set (Table 3 and Table S5). As Figure 5A shows, 72 and 1 genes were up-regulated exclusively in the reference strain and the *ΔatfA* mutant, respectively, while the numbers of exclusively down-regulated genes were 2 and 89.

This difference between the strains may reflect the difference between the two untreated cultures: Before stress treatment, 168 genes showed higher and only 1 lower transcription value in the mutant than in the reference strain. After stress treatment, only 34 genes had higher and 43 had lower transcription activity in the *atfA* gene deletion mutant than in the reference strain (Figure 5B). The divergent response found in the two strains may be the consequence of both strains aiming to set similar transcription profile of the “Ribosome biogenesis” genes under stress but they started from very different transcription levels (Figure 5; Table S5). 

The behavior of “Folate cycle” genes can also be explained in a similar manner. Cadmium stress up-regulated six genes (AN0495, AN1524, AN4776, AN5738, AN6108, AN7028) in the reference strain and only one (AN0495) in the *ΔatfA* mutant (Table 3 and Table S5), resulting in significant enrichment of up-regulated “Folate-cycle” genes in THS30, but not in TNJ92. All six genes showed lower transcriptional activity in the reference strain than in the *ΔatfA* mutant before stress treatment, which may explain the divergent response of the two strains (Figure S6 and Table S5).

#### 3.5.3. Transcriptional Activities of Certain Genes Are Not Properly Regulated in the Absence of AtfA

**Figure 6:**
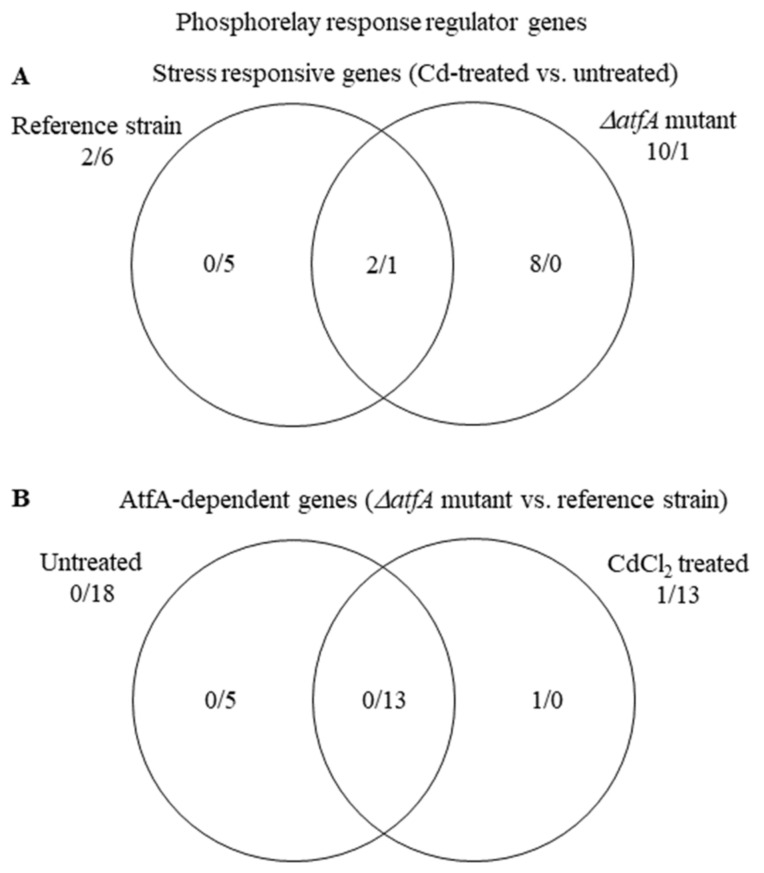
Distribution of stress responsive “Phosphorelay response regulator” genes between the THS30 reference strain and the TNJ92 *ΔatfA* mutant (**A**) as well as of the AtfA-dependent “Phosphorelay response regulator” genes between the untreated and CdCl_2_-treated cultures (**B**). Figures represent the number of up-regulated/down-regulated genes. Further data on the gene group are available in Table S5.

Among the 20 “Phosphorelay response regulator” genes, in untreated cultures, the majority (18) of the genes showed significantly smaller transcription in the TNJ92 mutant than in the THS30 reference strain (Table S5, Figure 6). Under stress, the mutant up-regulated 10 genes and the reference strain down-regulated 6 (Figure 6A), yet the difference between the two strains remained substantial (Figure 6B). The stress condition only reduced, but did not eliminate, the difference between the activities of “Phosphorelay response regulator” genes in the two strains.

**Figure 7:**
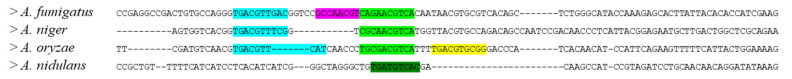
Evolutionary conserved putative AtfA binding site in the promoters of the *catA* ortholog genes.

In the above case, the mutant—in the absence of AtfA—appeared unable to appropriately regulate the studied gene group. Therefore, we investigated the presence of putative AtfA binding sites in the promoters of these genes. As it was, according to what our previous studies [23,24,25] expected, the promoters of the “Phosphorelay response regulator” genes contained significantly more putative AtfA binding sites than the reference gene groups did (Table 4 and Table S6). The AtfA dependence of catalase gene expressions also seems likely (Table 4 and Table S6), which concurs with previous studies [12,15,20,63] and the transcription of data of *catA* and *catB* (Table S5, Figure S7). Promoter analyses of the orthologs of *A. nidulans* “Phosphorelay response regulator” genes as well as *catA* and *catB* were also conducted in three other *Aspergillus* species (*A. fumigatus, A. niger,* and *A. oryzae*). In the case of the catalase genes, the numbers of the found putative binding sites in each species were similar to those recorded with *A. nidulans* (Table S6). Moreover, an evolutionary conserved binding site was also found in the promoter of *catA* orthologs in *A. fumigatus, A. niger,* and *A. oryzae* (Figure 7). In the case of the “Phosphorelay response regulator” genes, the orthologs of *A. niger* had significantly less putative binding sites than those of *A. nidulans* (Table S6). In the case of this gene group, no evolutionary conserved binding sites were recorded. It can be explained by the rather different biology of the four *Aspergillus* species, which increases the number of species-specific regulatory sequences and eradicates the evolutionary conserved ones. The weak conservation of the AtfA binding sites in the promoter of *A. nidulans catA* also supports this view (Figure 7).

Promoter regions were aligned with the MAFFT software (http://www.ebi.ac.uk/Tools/msa/mafft, accessed on 2 July 2021). Blue, purple, light green, and yellow highlight putative binding sites characterized with less than 5% maximum matrix dissimilarity rate (Promo tool). The dark green color highlights a putative AtfA binding site of *A. nidulans* where the dissimilarity rate for CRE-BP2 [T01017] (Promo tool) binding site is 13.5%. The following sequences, downloaded from AspGD, are presented: *A. fumigatus* Af293 *catA* promoter region between ATG-221 and ATG-109, *A. niger catA* promoter region between ATG-242 and ATG-144, *A. oryzae* RIB40 *catA* promoter region between ATG-236 and ATG-140, and *A. nidulans* FGSC A4 *catA* promoter region between ATG-197 and ATG-108.

#### 3.5.4. Alternative Stress Response Elements Can Increase the Difference between the Stress Responses of the Two Strains

**Figure 8:**
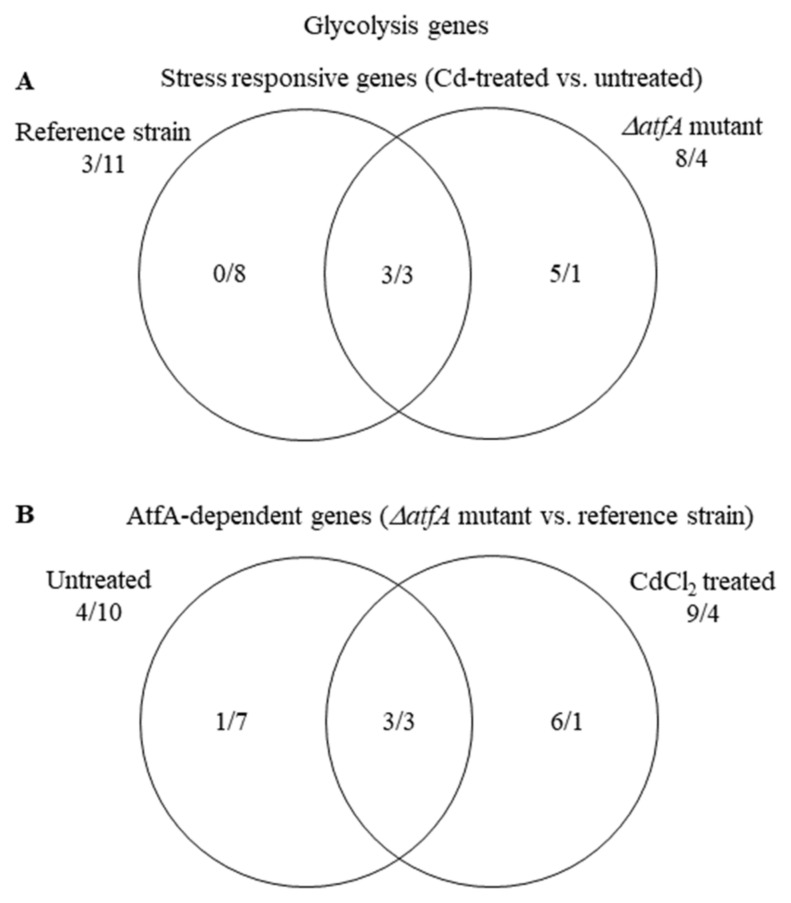
Distribution of stress responsive “Glycolysis” genes between the THS30 reference strain and the TNJ92 *ΔatfA* mutant (**A**) as well as of the AtfA-dependent “Glycolysis” genes between the untreated and CdCl_2_-treated cultures (**B**). Figures represent the number of up-regulated/down-regulated genes. Further data on the gene group are available in Table S5.

Both up-regulation and down-regulation of “Glycolysis” genes could have adaptive value during stress. Up-regulation of this biochemical pathway can support the protection against Cd^2+^ with increased ATP production. On the other hand, its down-regulation can adapt to the reduced growth rate under stress. “Glycolysis” genes were enriched in the down-regulated gene set of the THS30 reference strain and many of them were up-regulated in the TNJ92 mutant; however, their enrichment was not significant (Table S5, Figure 8A). Due to these changes, the ratio of genes showing higher/lower transcriptional activity in the TNJ92 mutant in comparison to the THS30 reference strain turned from 4/10 (untreated cultures) to 9/4 (stress treated cultures) (Table S5, Figure 8B).

In addition to its genotoxicity [61] cadmium can also disturb the activity of repair enzymes [64]. Therefore, similarly to “Glycolysis” genes, either up-regulation or down-regulation of “DNA repair” genes could be an adaptive response under cadmium stress. The ratio of genes with higher/lower transcriptional activity in the *ΔatfA* mutant than in the reference strain also turned to the opposite under stress, from 22/12 (untreated cultures) to 14/24 (cadmium-treated cultures) (Table S5). 

In the case of these gene groups, the two strains seem to apply two different strategies to adapt to the stress.

## 4. Discussion

### 4.1. Increased Cys Availability Is an Important Element of the CdCl_2_-Induced Stress Response

Genome-wide transcriptional changes induced by CdCl_2_ were studied in an *A. nidulans* model organism in order to gain deeper insight into the reasons of Cd^2+^ toxicity and the biochemical processes necessary to the detoxification of this heavy metal. Entering the cells, Cd^2+^ interacts with susceptible proteins (e.g., different metallo- or thiol-containing proteins) perturbing their activity and functions. It disturbs metal ion (*e.g.,* iron, zinc, copper) homeostasis, protein folding, DNS repair processes, and leads to oxidative stress, aggregation of nascent proteins, or increased mutation rate, which all affect severely the viability of cells [65,66,67,68]. Many of the observed transcriptional changes concur well with these consequences of cadmium treatment. Significant alterations in the transcription of DNA repair genes and up-regulation of the unfolded stress response genes together with several ER specific genes were observed (Table 2, Table 3 and Table S5). Down-regulation of Fe-S cluster biosynthesis genes as well as other mitochondrium specific genes encoding iron-dependent proteins (e.g., respiration and TCA cycle genes) (Table 2, Table 3 and Table S5) can be the consequence of disturbed iron homeostasis caused by Cd^2+^. Besides the altered transcription of the antioxidative enzyme genes (Table S5), the increased specific SOD activities and GSSG concentrations as well as the disturbed redox homeostasis (Figure 1, Table 1) demonstrate developing oxidative stress during the CdCl_2_ treatment. Up-regulation of specific SOD, catalase, and glutathione reductase activities induced by cadmium stress were also recorded previously in *A. nidulans* by Guelfi et al. [69]. Since cadmium is not a Fenton metal, it can induce oxidative stress only indirectly. Both the disturbed iron/mitochondrium homeostasis and the changes in the activity of ER as another important ROS-generating organelle [70,71] can be vital in this process.

The cell wall of *A. nidulans*, similarly to those of many other fungi, can bind a huge amount of Cd^2+^ [9,10,72,73], which prevents this toxic metal from entering the cells. This property of fungal biomass can be used both for bioleaching and biomining purposes in the industry [72,74,75]. Although cadmium stress affected the transcription of some genes involved in cell wall biosynthesis or degradation, significant enrichment of the up-regulated or down-regulated cell wall homeostasis genes was not observed (Table S5). This supports the view that the high Cd^2+^ (metal ion) binding capacity is an innate property of the fungal cell wall and no substantial cell wall remodeling is needed for the efficient binding. It is an important feature that increases the applicability of fungal biomass in biosorption based processes.

There are multiple ways in fungi to detoxify Cd^2+^ entering their cells. These include secreting Cd^2+^ by efflux pumps, binding Cd^2+^ with metallothioneins, and the formation and vacuolar accumulation of the bis(glutathionato)-cadmium complex [76]. Up-regulation of *crpA* (Tables S3 and S5) encoding a P-type ATPase by cadmium stress in our experiments concurs with the observation that deletion of this gene was accompanied with increased CdCl_2_ sensitivity of *A. nidulans* [9,46]. Transcriptome data did not provide clear evidence on the working of Cd^2+^ pumps other than CrpA in *A. nidulans*: Apart from *crpA*, only two other putative P-type ATPase genes (AN0318 and AN10367) were up-regulated by cadmium stress (Table S5). Of these, the AN10367 gene, like *crpA*, was also up-regulated in the TNJ92 mutant (Table S5). The AN0318 protein is an ortholog of *Neurospora crassa* Pma-1 and *S. cerevisiae* Pma1p H^+^-ATPase [77,78], while AN10367 is an ortholog of *S. cerevisiae* Spf1p and *S. pombe* Cta4p Ca^2+^ pumps, which are responsible for the calcium homeostasis of the ER [79,80]. The function of the two *A. nidulans* genes is waiting for experimental justification; however, according to the abovementioned data, CrpA seems to be the sole Cd^2+^ pump of *A. nidulans* under the tested conditions.

Although *crdA*, like many other genes encoding “Cys rich proteins”, was down-regulated by the cadmium stress (Table S5), two genes from this group (AN7290 and AN11757) showed up-regulation. AN7290, which was also up-regulated in TNJ92 mutant under cadmium stress (Table S5), is an ortholog of *S. cerevisiae* Rds3p and *S. pombe* Ini1p zinc cluster proteins involved in mRNA splicing [81,82]. The AN7290 protein is 12.3 kDa and consists of 108 amino acids including 12 Cys (AspGD). The AN11757 protein has no orthologs with known function. It is 11.4 kDa and consists of 100 amino acids including 13 Cys (AspGD). Further studies are needed to test whether these proteins function as metallothioneins under cadmium stress in *A. nidulans*. 

Ycf1p of *S. cerevisiae* is an ABC transporter involved in the transportation of bis(glutathionato)–cadmium complex into the vacuole [50]. Up-regulation of the *ycf1* ortholog AN7727 under cadmium stress (Table S5) supports the view that GSH dependent detoxification of this heavy metal is an important element of stress response under Cd^2+^ treatment in *A. nidulans*, similarly to other organisms from bacteria to human [51]. 

Fe-S cluster binding proteins and proteins with high Cys content are particularly sensitive to the presence of Cd^2+^ and/or the oxidative stress caused by Cd^2+^ [66,83]. To manage this problem, cells can down-regulate genes encoding nonessential Cys rich and Fe-S cluster binding proteins as well as they can eliminate the destroyed proteins and can re-synthetize those that are important for surviving this stress. Enrichment of the “Cys-rich protein” and “Fe-S cluster binding protein” genes within the down-regulated gene set as well as the enrichment of “Proteosomal degradation” and “Autophagy” genes within the up-regulated gene set were all observed (Table 3 and Table S5). Moreover, the up-regulation of the transsulfuration pathway, GSH degradation, Cys biosynthesis genes and Cys synthase activities, and down-regulation of Met cycle and Met salvage pathway genes (Table S5, Figure 3) suggest that cells increased Cys availability during cadmium stress. It concurs with the high Cys demand of the continuous re-synthesis of Cys rich and/or Fe-S cluster binding proteins. These data demonstrate that GSH is involved not only directly in Cd^2+^ detoxification, but also indirectly as an important intracellular Cys storage. During oxidative stress, the thiol-moiety of proteins are continuously oxidized to disulfide bridges and cells continuously reduce them back. This process substantially contributes to ROS elimination [84]. It is possible that a similar mechanism (i.e., replacing Cd^2+^-bound proteins with new ones) can help cells to collect and detoxify Cd^2+^.

### 4.2. Adaptation to the Missing AtfA under Unstressed Conditions Influences Substantially the CdCl_2_ Induced Stress Response

In addition to the THS30 reference strain, the CdCl_2_-induced stress response was also studied in the TNJ92 *ΔatfA* mutant to better understand how this transcription factor is involved in the regulation of stress responses. Previously, we found that deletion of *atfA* increased the stress sensitivity of *A. nidulans* against various oxidative agents [23,24,25], which concur with its reduced specific catalase activity recorded in this study (Table 1). Accordingly, the stress response of the *ΔatfA* mutant substantially differed from that of the reference strain [23,24,25]. In contrast, AtfA was not essential for the efficient stress response under CdCl_2_ stress: The growth reduction induced by the stress treatment in the THS30 reference strain did not differ significantly to that of the TNJ92 *ΔatfA* mutant either on the surface [25] or in submerged cultures (Figure 1A). There were no significant differences between the two strains when the physiological consequences (i.e., alterations in the GSH and GSSG concentrations, in the specific SOD, catalases and Cys synthase activities, or in the redox homeostasis) of the cadmium treatment were studied either (Figure 1B,C, Table 1), while in the case of the extracellular chitinase or *N*-acetyl-β-D-glucosaminidase activities, only mild differences were detected (Table 1). Moreover, the transcription of *atfA* was even down-regulated under CdCl_2_ stress in the reference strain (Table S3), suggesting that this transcription factor was not a key player in the regulation of stress responses in these experiments. Surprisingly, the genome-wide transcriptional changes induced by the cadmium stress treatment in the THS30 and TNJ92 strains were very different (Figure 2A). It demonstrates that AtfA is involved in the cadmium stress response, but cells can efficiently compensate the consequences of its absence. It is very unlikely that *A. nidulans* has “learnt” during its evolution how to survive CdCl_2_ stress without AtfA. Therefore, we should assume that stress responses cannot be rigid programs worked out in detail for lots of possible stress conditions. Instead, cells should have flexible regulatory mechanisms that can find a suitable stress response even under brand new conditions.

The differences between the cadmium stress responses of the two strains are linked to the (missing) AtfA and can help to better understand the function of this transcription factor. In our previous oxidative stress treatment experiments, the two strains showed different sensitivities to the applied stressors [23,24,25]. In spite of the two strains receiving the same treatments, the effect of the treatments was not the same because the decreased stress tolerance of the *ΔatfA* mutant increased the difference between the observed stress responses, making identification of the direct effects of AtfA deletion more difficult. In this respect, cadmium stress experiments are more favorable since the cadmium tolerance of the THS30 and TNJ92 strains are very similar. Even so, identification of potential targets of AtfA is still challenging. There are at least two possible reasons of the altered cadmium stress responses of the two strains:

(1) The transcriptome, and as a consequence, the physiology of the THS30 and TNJ92 strains, were different under unstressed conditions (Figure 2B). Cells with dissimilar physiology necessarily respond differently to the same stress treatment. As a consequence, gene sets with dissimilar transcription profiles at unstressed conditions have to respond to the stress in different ways to reach the same transcription profile (Figure 5 and Figure S6). The dissimilar physiological status of the untreated cultures can also lead to alternative stress responses (Figure 8). The small physiological/transcriptomic differences between the untreated cultures may also explain why gene sets respond in the same overall manner to the stress but by the regulation of partly different genes (Figure 3; Figure 4 and Figures S3–S5).

(2) Due to the missing AtfA, cells could not regulate properly certain genes. The improper behavior of these genes can also lead to the development of alternative stress response elements or to the development of the same stress response element in an alternative manner. Moreover, the improper regulation of AtfA-dependent genes is necessarily the primary source of the observed differences between the two strains under unstressed conditions. Unfortunately, based on only transcription data, due to strong indirect effects of the missing AtfA, it is difficult to identify genes directly regulated by this transcription factor. The “Phosphorelay response regulator” genes and the “Catalase” genes are our best candidates for this (Figure 6 and Figure S7). In addition to the data gained in previous investigations [12,15,20,23,24,25,63] and the observed transcriptional behavior of these genes (Table S5), the presence of putative AtfA binding sites in their promoters (Table 4 and Table S6) supports this view. Although the number of putative binding sites was large, none but one was evolutionary conserved for the four *Aspergillus* species tested (Table S6, Figure 7). Therefore, detailed molecular biological studies are needed to ascertain which species-specific putative binding sites have regulatory function in *A. nidulans*.

## Figures and Tables

**Table 1:** Specific activities of selected enzymes in CdCl_2_-treated and untreated cultures of *A. nidulans* THS30 (reference) and TNJ92 (*ΔatfA*) strains.

	THS30 Untreated	THS30 CdCl_2_ Treated	TNJ92 Untreated	TNJ92 CdCl_2_ Treated
Catalase (kat/kg protein)	4.2 ± 0.5	3.5 ± 0.4	1.3 ± 0.1 ^b^	1.2 ± 0.2 ^b^
SOD (kU/kg protein)	64 ± 11	138 ± 20 ^a^	73 ± 10	140 ± 18 ^a^
Cys synthase (μKat/kg protein)	55 ± 8	99 ± 15 ^a^	53 ± 12	95 ± 14 ^a^
Chitinase (U/L)	<0.003	<0.003	<0.003	0.005 ± 0.003 ^a,b^
*N*-Acetyl-β-D- -glucosaminidase (U/L)	0.056 ± 0.012	0.059 ± 0.012	0.061 ± 0.010	0.041 ± 0.008 ^a,b^
β-Glucosidase (U/L)	0.08 ± 0.02	0.08 ± 0.01	0.14 ± 0.03 ^b^	0.15 ± 0.03 ^b^

Mean ± SD calculated from 4 independent experiments are presented. ^a^—Significant difference (Student’s *t*-test, *p* < 0.05) between the appropriate CdCl_2_-treated and the untreated cultures. ^b^—Significant difference between the appropriate THS30 and the TNJ92 strains.

**Table 2:** Overview on the significantly enriched FunCat, GO, and KEGG pathway terms.

Number of Up- and Down-Regulated Genes		Significantly Enriched Terms ^a^ for	
		Up-Regulated Genes	Down-Regulated Genes
**Reference strain, Cd treated vs. Reference strain, untreated**			
1875	1985	ribosome biogenesis (54), rRNA synthesis (30), mRNA processing (47), tRNA synthesis (20), aminoacyl-tRNA-synthetases (25), enoyl-CoA hydratase activity (4), obsolete ATP catabolic process (29), proteosomal degradation (76), stress response (92), nutrient starvation response (20), unfolded protein response (34), protein folding (18), protein processing in endoplasmic reticulum (33)	translation (90), ribosome biogenesis (70), purin nucleotide/nucleoside/nucleobase metabolism (39), glycolytic process (9), pyruvate metabolism (17), tricarboxylic-acid pathway (29), aerobic respiration (58), generation of precursor metabolites and energy (34), porphyrin and chlorophyll metabolism (11), Fe/S binding (24), C-1 compound catabolism (9), tetrahydrofolate interconversion (4) sterol metabolic process (14), acetyl-CoA carboxylase activity (5), biosynthesis of secondary metabolites (123), positive regulation of sterigmatocystin biosynthetic process (9)
***ΔatfA* mutant, Cd treated vs. *ΔatfA* mutant, untreated**			
2482	2514	aminoacyl-tRNA-synthetases (20), proteosomal degradation (88), stress response (114), nutrient starvation response (23), protein folding and stabilization (47), ER to Golgi transport (27), protein processing in endoplasmic reticulum (40), regulation of autophagy (8)	mitotic cell cycle (21), organization of chromosome structure (102), DNA synthesis and replication (73), mRNA synthesis (55), translation (120), ribosome biogenesis (144), deoxyribonucleotide metabolism (12), tricarboxylic-acid pathway (32), aerobic respiration (60), generation of precursor metabolites and energy (28), Fe/S binding (28), sterol metabolic process (17), acetyl-CoA carboxylase activity (5), one-carbon metabolic process (8), sulfate assimilation (8)
***ΔatfA* mutant, untreated vs. Reference strain, untreated**			
2840	2643	cytoskeleton (9), translation (138), ribosome biogenesis (158), protein folding and stabilization (64), N-directed glycosylation, deglycosylation (26), protein processing (proteolytic) (44), non-vesicular ER transport (8), unfolded protein response (35), Fe/S binding (26), aerobic respiration (61), purin nucleotide/nucleoside/nucleobase metabolism (54), pyrimidine metabolism (35), histidine metabolic process (11), sulfate assimilation (8), tetrahydrofolate-dependent C-1-transfer (11), G-protein mediated signal transduction (25)	development of asco- basidio- or zygospore (58), cell wall (78), glycolysis/gluconeogenesis (16), starch and sucrose metabolism (18), fructose and mannose metabolism (11), valine, leucine and isoleucine degradation (11), lipid/fatty acid transport (48), stress response (97), heat shock response (20), osmotic and salt stress response (35). two-component signal transduction system (16), MAPKKK cascade (16), non-ribosomal peptide synthesis (16),
***ΔatfA* mutant, Cd treated vs. Reference strain, Cd treated**			
2487	2386	cytoskeleton (8), N-directed glycosylation, deglycosylation (23), ER to Golgi transport (31), proteasomal degradation (ubiquitin/proteasomal pathway) (61), energy generation (e.g., ATP synthase)(13), amino acid metabolism (83), biosynthesis of methionine (12), biosynthesis of threonine (6), biosynthesis of vitamins, cofactors, and prosthetic groups (60), purine nucleotide/nucleoside/nucleobase anabolism (17)	development of asco- basidio- or zygospore (56), mitotic cell cycle (17), DNA synthesis and replication (44), organization of chromosome structure (77), DNA repair (74), osmosensing and response (30), two-component signal transduction system (12), MAPK signaling pathway–yeast (15)

^a^—Table contains selected FunCat, GO and KEGG pathway terms significantly enriched in the up-regulated and down-regulated gene sets. The complete list of all the significantly enriched terms is available in Table S4. Figures presented in parenthesis after the terms represent the number of up-regulated or down-regulated genes belonging to the appropriate term in the particular comparison.

**Table 3:** Overview on significant enrichment of the studied gene groups.

Significantly Enriched Group ^a^ for		
Up-Regulated Genes	Down-Regulated Genes	Stress Responsive/AtfA-Dependent Genes ^b^
**Reference strain, Cd treated vs. Reference strain, untreated**		
Ribosome biogenesis genes, Cys, Met and GSH metabolism genes, Folate cycle genes, Glycolysis genes, Autophagy related genes, ER to Golgi vesicle-mediated transport genes, inp cluster	Ribosome biogenesis genes, Cys rich proteins genes, Glyoxylate pathway genes, Folate cycle genes, Fe-S cluster assembly genes, Squalene-ergosterol pathway genes, Zinc transporter genes, Microperfuranone (mic) cluster, AN1242 cluster	Ribosome biogenesis genes, Glyoxylate pathway genes, Cys, Met and GSH metabolism genes, Folate cycle genes, Glycolysis genes, Fe-S cluster assembly genes, Squalene-ergosterol pathway genes
***ΔatfA* mutant, Cd treated vs. *ΔatfA* mutant, untreated**		
Autophagy related genes, ER to Golgi vesicle-mediated transport genes, Phosphorelay response regulator genes, inp cluster, AN7884 cluster, AN7084 cluster	Ribosome biogenesis genes, Squalene-ergosterol pathway genes, Zinc transporter genes, Glyoxylate pathway genes, Folate cycle genes, Fe-S cluster assembly genes, DNA repair, Microperfuranone (mic) cluster	Ribosome biogenesis genes, Autophagy related genes, Zinc transporter genes, Cell wall biosynthesis, Glyoxylate pathway genes, Cys, Met and GSH metabolism genes, Fe-S cluster assembly genes
***ΔatfA* mutant, untreated vs. Reference strain, untreated**		
Ribosome biogenesis genes, Cys, Met and GSH metabolism genes, Folate cycle genes, Fe-S cluster binding protein genes, Antioxidant enzyme genes, ER to Golgi vesicle-mediated transport genes, AN6236 cluster, Penicillin cluster, xptA-containing cluster	Cell wall biosynthesis and degradation, Cell wall biosynthesis, Chitin synthesis, Cell wall integrity pathway genes, Autophagy related genes, Phosphorelay response regulator genes, Glycolysis genes, AN7084 cluster, AN10486 cluster, AN3612 cluster, AN12331 cluster, No PKS/NRPS backbone cluster 1, AN2924 cluster, AN1594 cluster, AN9005 cluster, Emericellamide (eas) cluster	Ribosome biogenesis genes, Cell wall biosynthesis, Antioxidant enzyme genes, Phosphorelay response regulator genes, Glycolysis genes
***ΔatfA* mutant, Cd treated vs. Reference strain, Cd treated**		
Cys, Met and GSH metabolism genes, Antioxidant enzyme genes, ER to Golgi vesicle-mediated transport genes, Glycolysis genes, AN6236 cluster, AN8504 cluster, No PKS/NRPS backbone cluster 3, xptA-containing cluster	Cell wall biosynthesis and degradation, Cell wall biosynthesis, Chitin synthesis, Glyoxylate pathway genes, “Excision repair” genes, Secondary metabolite cluster key genes, Phosphorelay response regulator genes, pkb cluster, AN7084 cluster, AN2924 cluster, AN9005 cluster, Emericellamide (eas) cluster, AN9314 cluster	Chitin synthesis, Cys, Met and GSH metabolism genes, Antioxidant enzyme genes, Phosphorelay response regulator genes

^a^—Table contains all the gene groups, defined in the Section 2.6. “Evaluation of RNAseq data” chapter, which were significantly enriched in the up-regulated and down-regulated gene sets. RNAseq data of the studied gene groups are available in Table S5. ^b^—In the first two comparisons stress responsive genes, while in the last two comparisons AtfA-dependent genes were studied. In the case of secondary metabolite cluster genes, enrichment was studied only in the up-regulated and down-regulated gene sets.

**Table 4:** Abundance of putative AtfA binding sites in selected gene groups.

Number of	Gene Group ^a^						
	A	B	C	D	E	F	G
Genes	20	21	5	21	16	13	9
Genes with binding site	12 (E–G) ^b^	7 (G) ^b^	2	7 (G) ^b^	3	2	0
Binding sites	20 (D–G) ^c^	10	2	8	3	2	0

^a^—A: Phosphorelay response regulator genes; B: P-type ATPase genes; C: Known and putative catalase genes; D: Glycolysis genes; E: Squalene-ergosterol pathway genes; F: Folate cycle genes; G: Asperfuranone (afo) cluster genes. ^b^—The ratio of genes with putative binding sites and genes without putative binding sites in the gene group is significantly higher than that is in the gene group(s) indicated in parentheses according to the Fisher’s exact test (*p* < 0.05). ^c^—The ratio of putative binding sites and genes in the gene group is significantly higher than that is in the gene group(s) indicated in parentheses according to the Mann–Whitney–Wilcoxon test (*p* < 0.05).

## Data Availability

The raw and processed transcriptome datasets were uploaded to the Gene Expression Omnibus database (GEO; http://www.ncbi.nlm.nih.gov/geo/, accessed on 2 July 2021) with the following accession number: GSE166128.

## Supplementary Materials

The following are available online at https://www.mdpi.com/article/10.3390/microorganisms9071433/s1, **Figure S1**: Principal component analysis (PCA) of the transcriptomes of CdCl_2_-treated and untreated *A. nidulans* THS30 (reference strain) and TNJ92 (*ΔatfA* mutant) cultures, Figure S2: Correlation between RNAseq and RT-qPCR data, Figure S3: Changes in the Cys and Met metabolism under CdCl_2_ treatment in the *A. nidulans* TNJ92 *ΔatfA* mutant, Figure S4: Distribution of stress responsive “ER to Golgi vesicle-mediated transport” genes between the THS30 reference strain and the TNJ92 *ΔatfA* mutant (A) as well as of the AtfA-dependent “ER to Golgi vesicle-mediated transport” genes between the untreated and CdCl_2_-treated cultures (B), Figure S5: Distribution of stress responsive “Cell wall biosynthesis and degradation” genes between the THS30 reference strain and the TNJ92 *ΔatfA* mutant (A) as well as of the AtfA-dependent “Cell wall biosynthesis and degradation” genes between the untreated and CdCl_2_-treated cultures (B), Figure S6: Changes in the transcription of selected “Folate cycle” genes, Figure S7: Changes in the transcription of genes encoding known and putative catalases, Table S1: RT-qPCR primer pairs used in the study, Table S2: Read mapping statistics, Table S3: RT-qPCR data of the selected genes, Table S4: Significantly enriched FunCat, GO, and KEGG pathway terms. Table S5: RNAseq data and the results of enrichment analyses of the studied gene groups, Table S6: Results of the promoter analyses.
