# Supplementary material for: AtfA-Independent Adaptation to the Toxic Heavy Metal Cadmium in Aspergillus nidulans
Source: Microorganisms. 2021 Jul 2;9(7):1433. doi: 10.3390/microorganisms9071433 (PMC8307709; doi:10.3390/microorganisms9071433)
Supplement: Supplementary file 1 [file microorganisms-09-01433-s001.zip › Table S1.pdf]

**Table S1** RT-qPCR primer pairs used in the study.

| Gene | Foward primer | Reverse primer |
|---|---|---|
| AN0447 | 5′-GACTTCCCTACCTCCTTCTTG-3′ | 5′-GCTCCACTCTTTTCCACGG-3′ |
| AN1092 (*dugC*) | 5′-ACTGCGAAAACTTGGAACGG-3′ | 5′-CTAAGAACAATGCGAATGCCC-3′ |
| AN1513 (*cysF*) | 5’-GGGGAAGGGGTTACTTTGC-3′ | 5′-TAGAGCCACGCCAGAGATTG-3′ |
| AN1800 (*tcsB*) | 5′-TCATACAATCCGCCTTACAGC-3′ | 5′-ATCCCAATCCTTCATCCCC-3′ |
| AN2508 | 5′-GGACCAAGGAACACGACC-3′ | 5′-CGCATCAGCCCCAATCAAG-3′ |
| AN2581 (*hk-8-1*) | 5’-TGCTTCCTCAGTCCTCTC-3′ | 5′-ACAAATCCCGTCCTCCTTAG-3′ |
| AN2911 (*atfA*) | 5′-ACCGATACCCCCGATTAC-3′ | 5′-AAGCCCCTTCCGATACTG-3′ |
| AN3117 (*crpA*) | 5′-CTTCGTTGGTGGCGGATTAG-3′ | 5′-TCGGGTGATTGCTGCTTTC-3′ |
| AN3150 (*gcsA*) | 5’-AGGAGGGAGGTAGCAAAAG-3’ | 5’-CAGATGGAGGGTAATAAGGC-3’ |
| AN4113 | 5′-TTCTCGCCAGCATCTTCGC-3′ | 5′-AGCCGTAGTTCGTCGTCAG-3′ |
| AN4271 | 5′-ATGGTGCTCTCAGTTTGGTTG-3′ | 5′-GTGTCAATGCCCGTCGTTTTG-3′ |
| AN4376 (*gdhA*) | 5′-CGGCAAGTTCACCTACATCC-3′ | 5′-ACCCTCAGCAATATAGCGG-3′ |
| AN4809 (*gtaA*) | 5’-GACGCCGCTCCTATTCTCTG-3’ | 5’-ATTGCCTCTCGCTGGGTTAG-3’ |
| AN5134 (*gltA*) | 5′-TTACGGCGAGGGAAACAAG-3′ | 5′-CGAGAGTGAACGAGAGCAAAG-3′ |
| AN6542 (*actA*) | 5′-GAAGTCCTACGAACTGCCTGATG-3’ | 5′-AAGAACGCTGGGCTGGAA-3’ |
| AN6820 (*hk-8-3*) | 5′-GGCAGCGATGAGGACATTC-3′ | 5′-GGAGAGGGAGACATAGTGG-3′ |
| AN6838 (*tubC*) | 5’-CGGAAACTCGCCGTCAATAT-3’ | 5’-GGGCAAACCCGACAATAA-3’ |
| AN7103 (*cshA*) | 5′-GAGTGGGACGGGAAGAATG-3’ | 5′-GGTTACGATGGGAGACAGC-3’ |
| AN7729 | 5’-ACCTCGTTCCAACAATACGC-3’ | 5’-AACCCGAACAGAGCAACAAC-3’ |
| AN7945 (*hk2*) | 5′-CGATGTCACTTACGCCGC-3′ | 5′-AAACCGCTCTTGCTGCTG-3′ |
| AN8692 (*prxA*) | 5′-CTGGACTGAGGAGAAGGG-3′ | 5′-CAAGGACGGCAACAACATCG-3′ |
| AN8803 (*rodA*) | 5’-CAACAAGGGCAACAGCAAC-3’ | 5’-GAGGACAGCAACATCAAGC-3’ |
| AN9397 (*hacA*) | 5’-AGACGAAGAATGTGGTGGC-3’ | 5’-ACGCTGAAGAAGGAACTGG-3’ |
| AN10220 (*ccp1*) | 5′-GCGACCAAGAACCAAGACC-3′ | 5′-AACCAACAGGCGGAAAAACTC-3′ |
| AN10584 | 5′-GGTCTCTGTTTCGCTCTCTG-3′ | 5′-ACGGTTTGCCTCTTCATCATTC-3′ |
