# Supplementary material for: AtfA-Independent Adaptation to the Toxic Heavy Metal Cadmium in Aspergillus nidulans
Source: Microorganisms. 2021 Jul 2;9(7):1433. doi: 10.3390/microorganisms9071433 (PMC8307709; doi:10.3390/microorganisms9071433)
Supplement: Supplementary file 1 [file microorganisms-09-01433-s001.zip › Table S2.pdf]

Table S2 Read mapping statistics.

Sample	Number of sequenced reads	Aligned reads (%)
Reference strain, untreated, 1	11 568 834	79.98
Reference strain, untreated, 2	14 894 686	88.54
Reference strain, untreated, 3	15 360 372	81.30
Reference strain, $CdCl_2$ treated, 1	14 327 487	80.41
Reference strain, $CdCl_2$ treated, 2	15 640 405	82.04
Reference strain, $CdCl_2$ treated, 3	14 210 634	82.39
ΔatfA mutant, untreated, 1	15 698 460	88.04
ΔatfA mutant, untreated, 2	14 517 442	88.41
ΔatfA mutant, untreated, 3	16 011 236	90.19
ΔatfA mutant, $CdCl_2$ treated, 1	14 219 473	91.1
ΔatfA mutant, $CdCl_2$ treated, 2	14 747 338	89.53
ΔatfA mutant, $CdCl_2$ treated, 3	15 069 078	90.16
