# Supplementary material for: AtfA-Independent Adaptation to the Toxic Heavy Metal Cadmium in Aspergillus nidulans
Source: Microorganisms. 2021 Jul 2;9(7):1433. doi: 10.3390/microorganisms9071433 (PMC8307709; doi:10.3390/microorganisms9071433)
Supplement: Supplementary file 1 [file microorganisms-09-01433-s001.zip › Figures S1-S7.pdf]

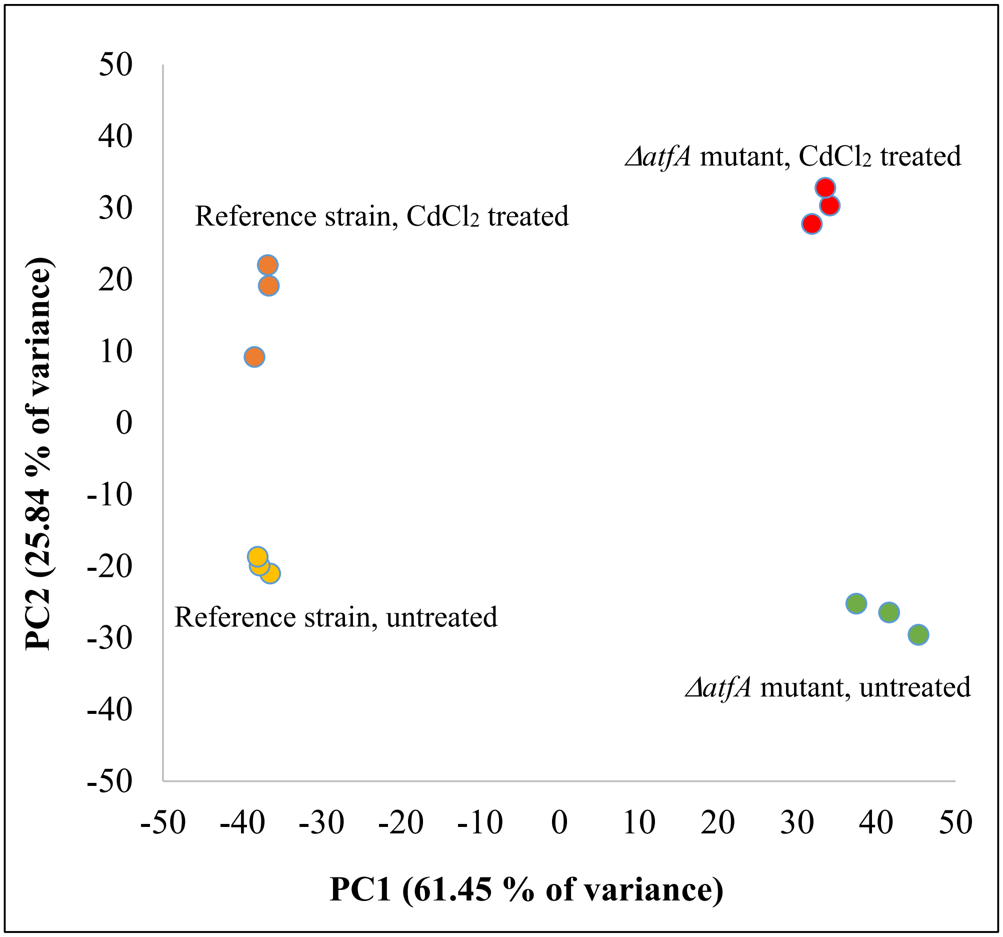

Figure S1 Principal component analysis (PCA) of the transcriptomes of $CdCl_2$-treated and untreated *A. nidulans* THS30 (reference strain) and TNJ92 (*ΔatfA* mutant) cultures.

PCA was based on "regularized log" values using the "rlog" function from the DESeq2 package (Love et al, 2014).

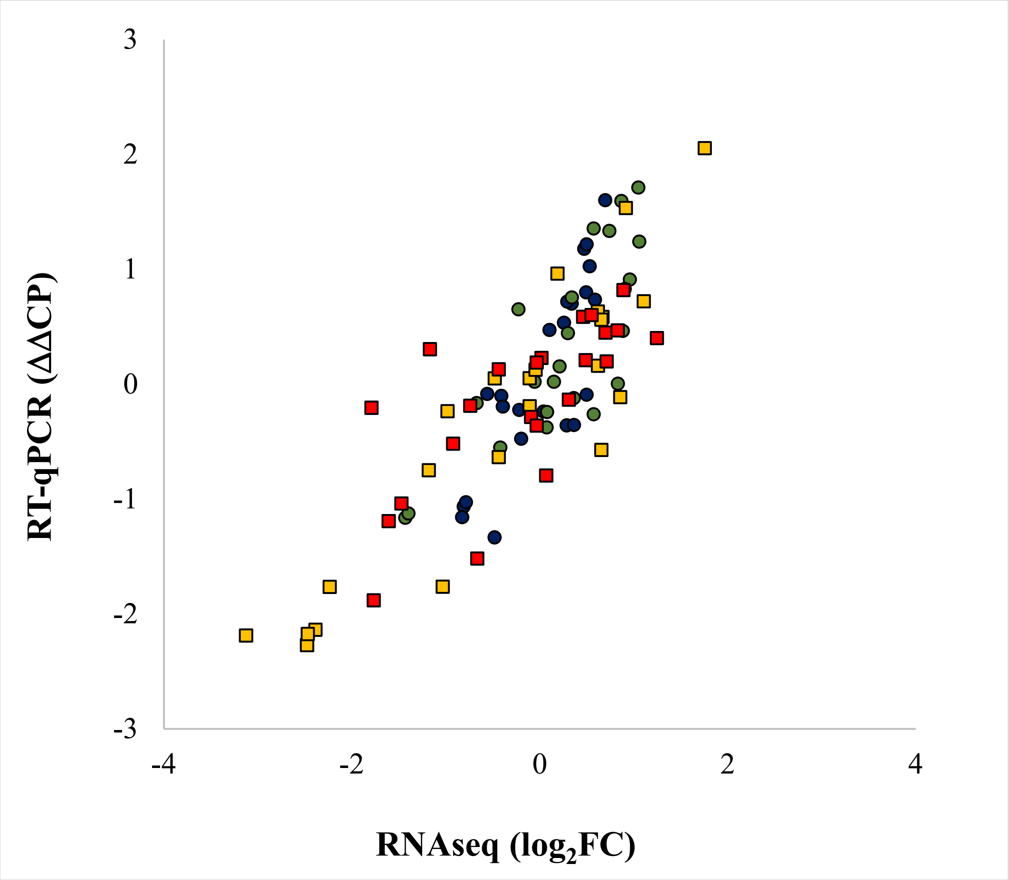

Figure S2 Correlation between RNAseq and RT-qPCR data.

All RT-qPCR data are available in Table S3. The Pearson's correlation coefficients were as follows: 0.83 with data of $CdCl_2$-treated vs. untreated cultures of the THS30 strain (●), 0.79 with data of $CdCl_2$-treated vs. untreated cultures of the TNJ92 mutant (●), 0.91 with data of TNJ92 mutant vs. THS30 under untreated conditions (■) and 0.72 with data of TNJ92 mutant vs. THS30 under $CdCl_2$-treated conditions (■).

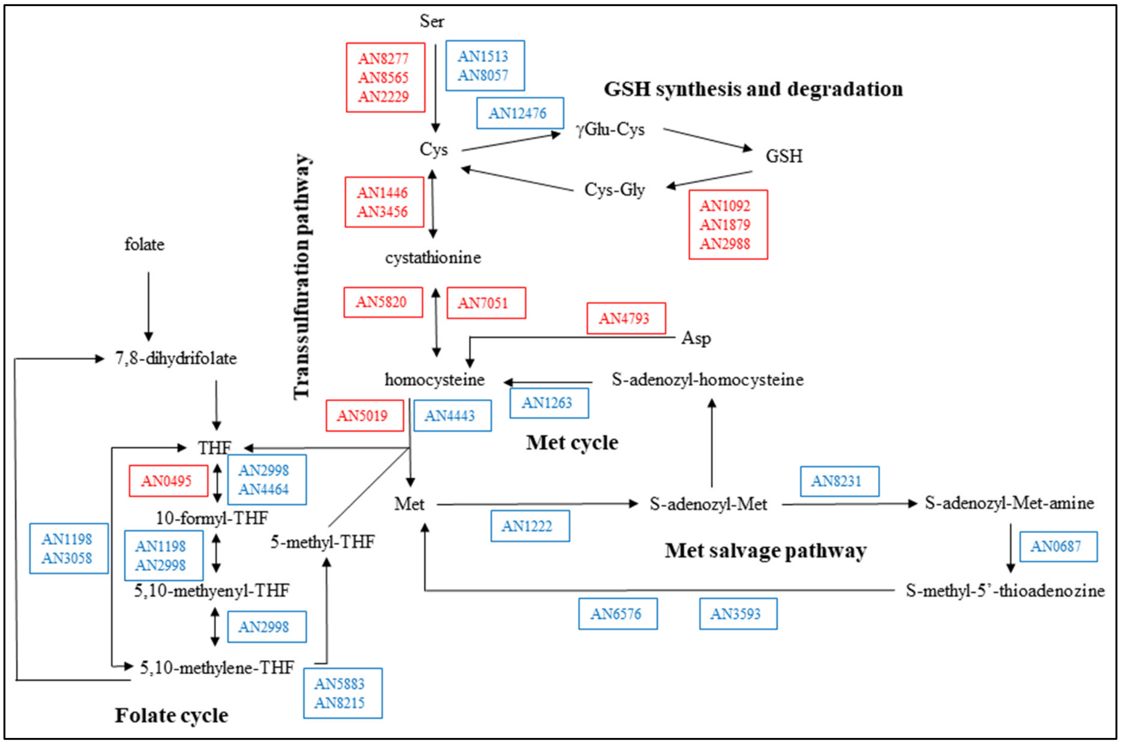

Figure S3 Changes in the Cys and Met metabolism under $CdCl_2$ treatment in the *A. nidulans* TNJ92 *ΔatfA* mutant.

Up-regulated genes are marked with red, down-regulated genes with blue.

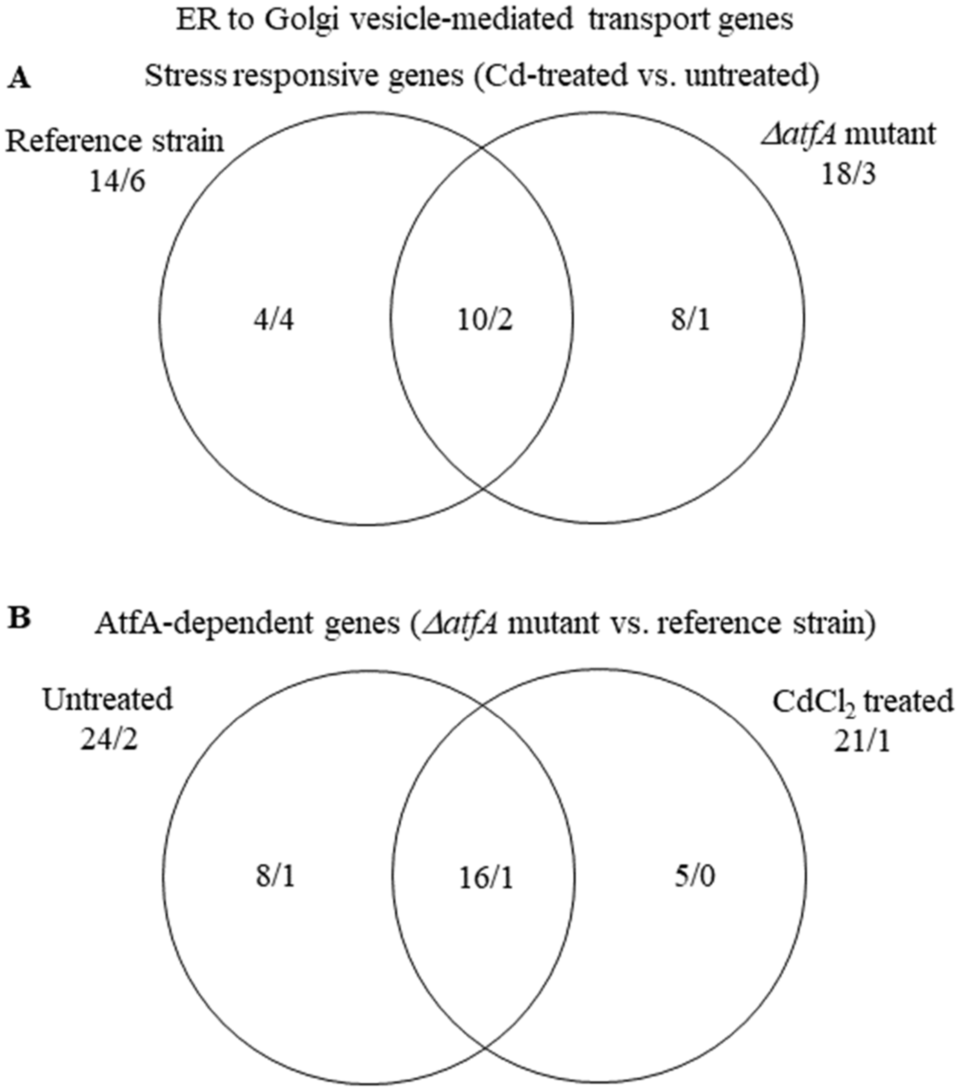

Figure S4 Distribution of stress responsive "ER to Golgi vesicle-mediated transport" genes between the THS30 reference strain and the TNJ92 *ΔatfA* mutant (A) as well as of the AtfA-dependent "ER to Golgi vesicle-mediated transport" genes between the untreated and $CdCl_2$ treated cultures (B).

Figures represent the number of up-regulated/down-regulated genes. Further data on the gene group are available in Table S5.

Cell wall biosynthesis and degradation genes

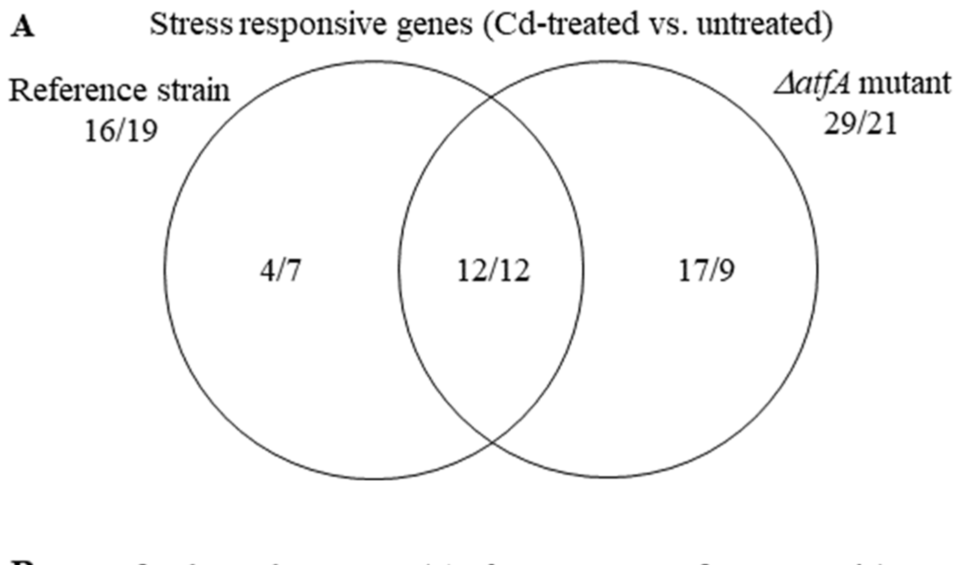

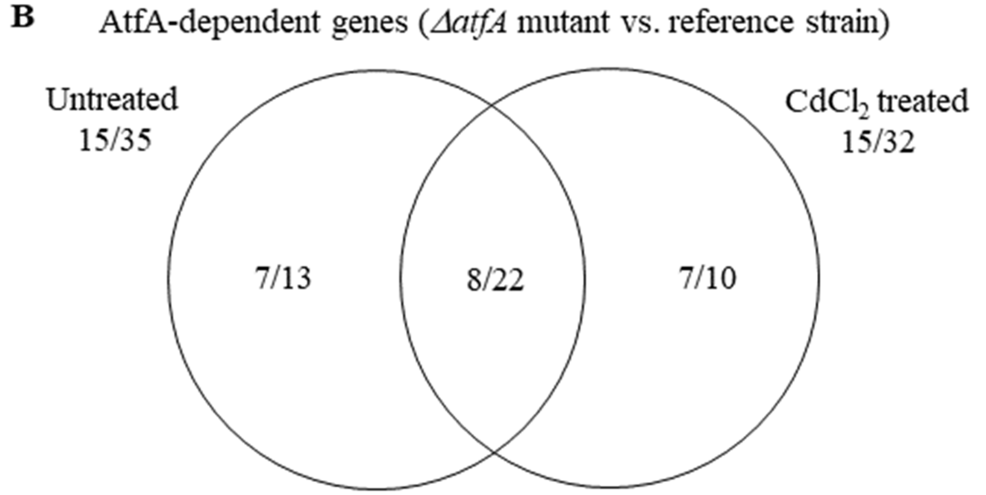

Figure. S5 Distribution of stress responsive "Cell wall biosynthesis and degradation" genes between the THS30 reference strain and the TNJ92 *ΔatfA* mutant (A) as well as of the AtfA-dependent "Cell wall biosynthesis and degradation" genes between the untreated and $CdCl_2$ treated cultures (B).

Figures represent the number of up-regulated/down-regulated genes. Further data on the gene group are available in Table S5.

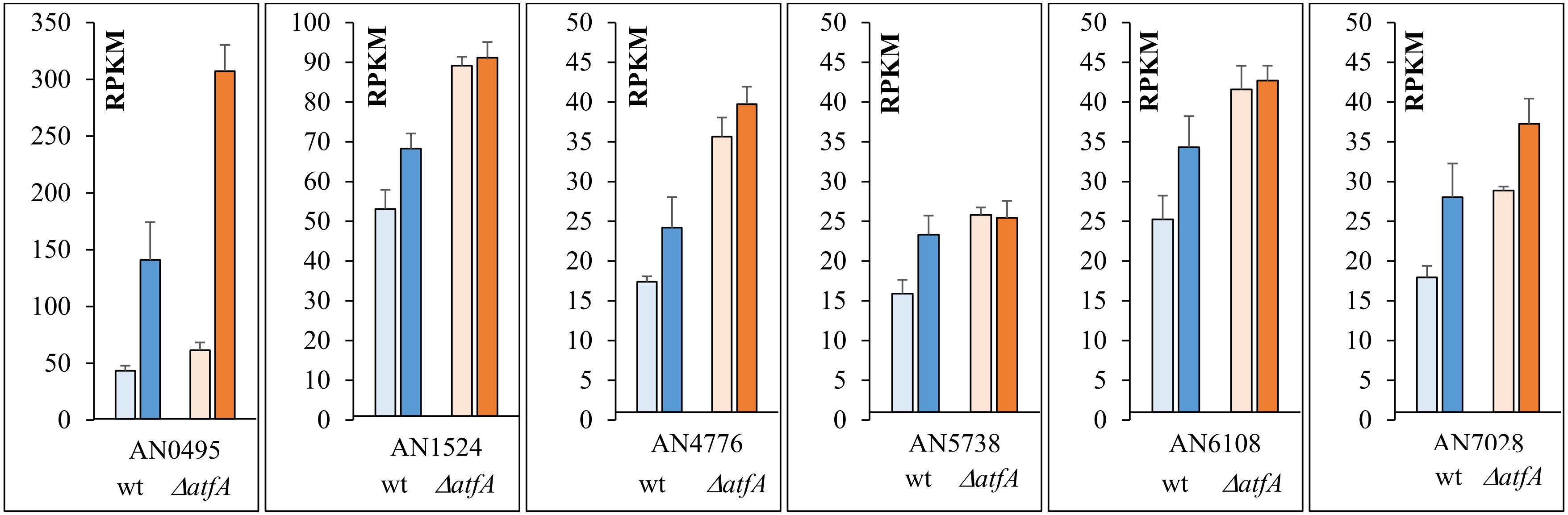

Figure S6 Changes in the transcription of selected "Folate cycle" genes.

The mean RPKM values of the THS30 reference strain (wt; blue) and the TNJ92 mutant (*ΔatfA*; orange) under untreated (light colors) and $CdCl_2$ treated (dark colors) conditions are presented. Only data of genes were up-regulated in the reference strain after stress treatment are presented. The AN0495 gene showed up-regulation in the mutant as well. Error bars represent S.D. values. For further details of the genes see Table S5.

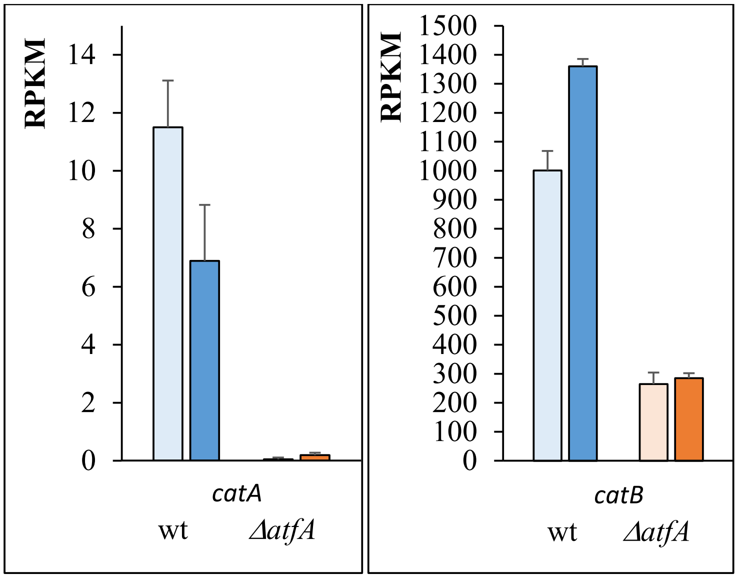

Figure S7 Changes in the transcription of *catA* and *catB* catalase genes.

The mean RPKM values of the THS30 reference strain (wt; blue) and the TNJ92 mutant (*ΔatfA*; orange) under untreated (light colors) and $CdCl_2$ treated (dark colors) conditions are presented. Error bars represent S.D. values. For further details of the genes see Table S5.
